# What can cardiac magnetic resonance do for cardiologists in China?

**DOI:** 10.12669/pjms.295.3823

**Published:** 2013

**Authors:** Rui Xia, Fabao Gao, Yucheng Chen, Chunhua Wang, Bing Zhang

**Affiliations:** 1 Rui Xia, PhD, Department of Radiology, West China Hospital, Sichuan University, No. 37 Guoxuexiang, 610041 Chengdu, China.; 2 Fabao Gao, MD, PhD, Department of Radiology, West China Hospital, Sichuan University, No. 37 Guoxuexiang, 610041 Chengdu, China.; 3 Yucheng Chen, MD, PhD, Department of Cardiovascular, West China Hospital, Sichuan University, No. 37 Guoxuexiang, 610041 Chengdu, China.; 4 Chunhua Wang, MM, Department of Radiology, West China Hospital, Sichuan University, No. 37 Guoxuexiang, 610041 Chengdu, China.; 5 Bing Zhang, MM, Department of Radiology, West China Hospital, Sichuan University, No. 37 Guoxuexiang, 610041 Chengdu, China.

Cardiovascular magnetic resonance (CMR) is well-established and increasingly used in clinical practice for the diagnosis of myocardial diseases.^1^ CMR is able to quantitatively evaluate left ventricular (LV) function, structural abnormalities of the myocardial tissue including edema, infarct size, and myocardial salvage in a single examination.^2^ CMR with contrast-enhanced protocols for tissue characterisation have dramatically changed the non-invasive work-up of patients with suspected or known cardiomyopathy. Although CMR initially focused on the in vivo identification of myocardial infarction, recent work highlighted the advantage of CMR to provide more detailed in vivo tissue characterisation to help establish a differential diagnosis of the underlying aetiology.^3^

CMR have been performed routinely in some hospitals in the USA and other developed countries. However, there are few hospitals where CMR is performed for clinical practice in China. In our hospital, 285 (146 in-patient, 139 out-patient) patients with heart disease who underwent the comprehensive CMR (3.0T MRI, MAGNETOM Trio, Siemens Medical Solutions, Erlangen, Germany) were admitted in cardiovascular department from January 2011 to April 2013. All of these patients were diagnosed with CMR (including T2 weighted imaging, perfusion, delayed enhancement and heart function measurement). Interestingly, the most common disease was not myocardial infarction (60, 21.1%) but hypertrophic cardiomyopathy (70, 24.6%) in these patients. Other myocardial diseases included dilated cardiomyopathy (51, 17.9%), Myocarditis (30, 10.5%), hypertension (24, 8.4%), amyloidosis (7, 2.5%), restricted cardiomyopathy (6, 2.1%), arrhythmogenic right ventricular cardiomyopathy (3, 1.1%), hyperthyroid heart disease (2, 0.7%), left ventricle non-compaction (1, 0.4%), fabry disease (1, 0.4%) and other uncatelogued cardiomyopathy (30, 10.5%).

 Although CMR can offer a unique non-invasive tool which should be integral part of the clinical work-up of a cardiomyopathy,^3^ the implementation is mainly limited by its long acquisition time and high costs in China. Further work needs to be done to establish CMR as an essential diagnostic tool for cardiologists.
